# RANK/RANKL/OPG system in the intervertebral disc

**DOI:** 10.1186/s13075-017-1332-y

**Published:** 2017-06-02

**Authors:** Norihiko Takegami, Koji Akeda, Junichi Yamada, Tomohiko Sano, Koichiro Murata, Jenny Huang, Koichi Masuda, Akihiro Sudo

**Affiliations:** 1 Department of Orthopaedic Surgery, Mie University Graduate School of Medicine, 2-174 Edobashi, Tsu City, Mie 514-8507 Japan; 2 Department of Orthopaedic Surgery, University of California, San Diego, 9500 Gilman Dr, La Jolla, 92093-0863 USA

**Keywords:** Receptor activator of nuclear factor kappa B, Receptor activator of nuclear factor kappa B ligand, Osteoprotegerin, Intervertebral disc, Cartilaginous endplate, Proinflammatory cytokine

## Abstract

**Background:**

The receptor activator of NF-κB ligand (RANKL), a member of the TNF ligand superfamily, is known to regulate bone metabolism. The expression of each component of the RANK/RANKL/osteoprotegerin (OPG) system in the intervertebral disc (IVD) has not been examined in detail. The purposes of this study were to examine the expression of the RANK/RANKL/OPG system and to evaluate the function of RANKL in the matrix metabolism of the rat IVD.

**Methods:**

Sprague-Dawley, 12-week-old, male rats were used in this study. Anulus fibrosus (AF), nucleus pulposus (NP) and cartilaginous endplate (CEP) cells isolated from dissected thoracolumbar discs were monolayer-cultured. RANK/RANKL/OPG expression in rat IVDs was examined using real-time polymerase chain reaction (PCR) and immunohistochemical analysis (cultured cells and IVD tissues). To examine the effect of interleukin-1β (IL-1β) stimulation on the mRNA levels of *RANK*, *RANKL* and *OPG*, the cells were cultured with or without recombinant human IL-1β (rhIL-1β). To evaluate the effect of RANKL on the mRNA expression of catabolic factors (*IL-1*β, matrix metalloproteinase-3 (*MMP-3*) and *MMP-13*), the cells were cultured with RANKL in the presence or absence of rhIL-1β. The immunohistochemical expression of this system was also evaluated using human IVD tissues with different grades of degeneration.

**Results:**

mRNA expression levels of *RANK*, *RANKL*, and *OPG* were clearly identified in AF, NP and CEP cells. Immunoreactivity to RANK, RANKL and OPG was distributed in the cell membranes and/or cytoplasm of the three types of cells. The mRNA level of *RANKL* was significantly upregulated by treatment with rhIL-1β of the three types of cells. Treatment with RANKL without rhIL-1β did not induce significant effects on the mRNA expression of catabolic factors by AF, NP and CEP cells. However, the expression was significantly upregulated by stimulation with RANKL in the presence of rhIL-1β. There was a general trend for more RANK/RANKL/OPG-positive cells in human IVD tissues in an advanced stage of degeneration compared to an early stage.

**Conclusions:**

Our study showed the possibility that the RANK/RANKL/OPG system may play a part in the process of intervertebral disc degeneration.

## Background

The vertebral column complex consists of ventrally located vertebral bodies and intervening intervertebral discs (IVDs) that are closely attached by cartilaginous endplates (CEPs). The IVD is composed of a central gelatinous nucleus pulposus (NP) and a surrounding fibrous anulus fibrosus (AF). Although the exact mechanism of IVD degeneration remains unknown, the biochemical changes typical of the degenerative IVD are known to include progressive decreases in proteoglycan and collagen type II contents with subsequent dehydration and increased content of collagen type I, which leads to tissue fibrosis [1]. Interleukin-1 (IL-1) and tumor necrosis factor-alpha (TNF-α) are thought to significantly affect matrix homeostasis during IVD degeneration. An in vitro study showed that IL-1 and TNF-α affect both anabolic and catabolic pathways by stimulating the production of nitric oxide, matrix metalloproteinases (MMPs) and aggrecanases and finally result in the decrease of proteoglycan content by both AF and NP cells [2–6].

Receptor activator of nuclear factor kappa B ligand (RANKL) is a member of the TNF ligand superfamily that is known to regulate bone metabolism [7, 8]. RANKL and its receptor (RANK) have been shown to play crucial roles in osteoclast differentiation and activation. The binding of RANKL to RANK activates TNF receptor-associated factor (TRAF) 6, which stimulates the expression of proinflammatory cytokines through nuclear factor kappa B (NF-κB) pathways [9]. Osteoprotegerin (OPG) is a soluble decoy receptor for RANKL that prevents it from binding to RANK. RANKL interacts with its receptor, which is expressed on osteoclast precursors, to induce differentiation and activation of osteoclasts [8]. The RANK-RANKL signal has been associated with bone matrix catabolism by osteoclasts [10–12]. It has recently been reported that the RANK/RANKL/OPG system is also expressed by human articular cartilage [13, 14]; the relevance of this system to the progression of osteoarthritis is controversial. More recently, RANKL [15, 16] and OPG [17] have been shown to be expressed by the human IVD, and are considered to be associated with the progression of IVD degeneration.

We hypothesized that expression of the RANK/RANKL/OPG system is associated with IVD degeneration. The purposes of this study were: (1) to examine the mRNA and immunohistochemical expression of the RANK/RANKL/OPG system in the rat IVD, including the CEP; (2) to examine the expression of the RANK/RANKL/OPG system under stimulation by IL-1β; (3) to evaluate the effect of RANKL on the expression of catabolic factors with and without proinflammatory stimulation and (4) to examine the expression of the RANK/RANKL/OPG system in the human IVD with different stages of degeneration.

## Methods

Sprague-Dawley, 12-week-old, male rats were used in this study, with institutional animal care committee approval.

### Cell preparation and culture conditions

IVDs were harvested from 10 rat spines for each series of cell culture experiments. The AF, NP and CEP were carefully dissected from thoracolumbar discs. IVD cells were isolated by sequential enzyme digestion with 0.4% Pronase (Calbiochem, La Jolla, CA, USA) for 1 hour (AF, CEP) or 30 minutes (NP), followed by 0.025% Collagenase P (Roche Applied Science, Mannheim, Germany) for 3 hours (AF), 30 minutes (NP) or 1 hour (CEP) in a 5% CO_2_, 95% air incubator at 37 °C. Following enzyme digestion, the suspension was filtered through a 40-μm mesh (BD Falcon, Franklin Lakes, NJ, USA) for AF cells or 70-μm mesh for NP and CEP cells. The filtered cells were first washed with Dulbecco’s modified Eagle’s medium and Ham’s F-12 medium (DMEM/F12; Gibco, Palo Alto, CA, USA) and primary culture was begun. Isolated cells were cultured in monolayer in 4-chamber slides (BD Falcon) for immunohistochemical analysis at 1.4 × 10^4^ cells/mL or 6-well tissue culture plates (BD Falcon) at 4.0 × 10^4^ cells/mL, with 5% CO_2,_ 95% air in complete medium (DMEM/F12 containing 10% fetal bovine serum (FBS; Biological Industries, Kibbutz Beit Haemek, Israel), 25 μg/mL ascorbic acid (Sigma-Aldrich) and 50 μg/mL gentamicin (Gibco)]. The medium was changed every third day. Primary cultured cells (in cumulative population doublings) were used in all the experiments conducted in this study.

### The effect of IL-1β stimulation on mRNA levels of *RANK*/*RANKL*/*OPG*

Following 7 (AF, CEP) or 10 days (NP) of monolayer pre-culture, the AF, NP or CEP cells were cultured in serum-free medium for 24 hours. The cells were then cultured with or without recombinant human IL-1β (rhIL-1β; R&D Systems, Minneapolis, MN, USA) at 0.01, 0.1, 1.0 or 10 ng/mL in DMEM/F12 containing 0.3% FBS, for an additional 24 hours.

### The effect of RANKL on the mRNA expression of anabolic and catabolic factors with or without IL-1β

After 7 (AF, CEP) or 10 days (NP) of monolayer pre-culture, all the cells were cultured in serum-free medium for 24 hours. The cells were then cultured in DMEM/F12 containing 0.3% FBS, for an additional 24 hours with or without 10 or 100 ng/mL recombinant human RANKL (rhRANKL; Wako Pure Chemical, Osaka, Japan) in the presence or absence of rhIL-1β (1.0 ng/mL).

### RNA isolation

Total cellular RNA was extracted from rat AF, NP and CEP cells in monolayer culture using Isogen (Nippon Gene, Toyama, Japan), according to the manufacturer’s instructions. Total RNA was reverse-transcribed using the first-strand Complementary DNA (cDNA) synthesis kit (Roche Applied Science) with the DNA thermal cycler (Veriti; Applied Biosystems, Foster City, CA, USA), according to the manufacturer’s protocol.

### Quantitative real-time polymerase chain reaction (PCR)

Following treatment with IL-1β and/or RANKL, the resultant cDNA (in triplicate) was amplified for the following target genes: *RANK*, *RANKL*, *OPG*, *IL-1*β, *MMP-3* and *MMP-13*. Inventoried (ready-made) primers corresponding to target genes were used in this study (Table 1: TaqMan Gene Expression Assays, Applied Biosystems). Real-time PCR was performed using the ABI PRISM 7000 Sequence Detection System (Applied Biosystems). PCRs were carried out in duplicate with 1 cycle at 50 °C for 2 minutes, 1 cycle at 95 °C for 10 minutes and 40 cycles at 95 °C for 15 seconds and at 60 °C for 1 minute. The assay was calibrated using *18S ribosomal RNA *(*rRNA*) as an internal control.

**Table 1:** Primers for real-time polymerase chain reaction (PCR)

Genes	Assay ID^a^	Size (bp)
*RANK*	Rn04340164_m1	63
*RANKL*	Rn00589289_m1	69
*OPG*	Rn00563499_m1	75
*IL-1*β	Rn00580432_m1	74
*MMP-3*	Rn00591740_m1	67
*MMP-13*	Rn01448192_m1	93
*18S rRNA*	Hs99999901_s1	187

*RANK* receptor activator of nuclear factor kappa, *RANKL* rank ligand, *OPG* osteoprotegerin, *MMP* matrix metalloproteinase, *rRNA* ribosomal RNA. ^a^TaqMan Gene Expression Assays (Applied Biosystems)

### Immunohistochemical analysis of rat IVD cells

Rat AF, NP and CEP cells were monolayer-cultured on 4-chamber slides for 4 days. The cells were fixed in 4% paraformaldehyde, treated with a blocking solution containing 10% skim milk, and permeabilized in 0.1% Triton X-100 (NACALAI TESQUE Inc., Kyoto, Japan), then incubated with anti-RANK (ab12008, 1:500; Abcam, Cambridge, UK), anti-RANKL (ab9957, 1:500; Abcam) or anti-OPG (sc- 8468, 1:50; Santa Cruz Biotechnology, Santa Cruz, CA, USA) antibodies overnight at room temperature. For isotype control, the cells were incubated with mouse isotype-matched immunoglobulin G (IgG; Dako, Glostrup, Denmark) instead of the primary antibodies. Secondary Alexa 488-conjugated anti-mouse IgG (1:400; Molecular Probes, Eugene, OR, USA), anti-rabbit IgG or anti-goat IgG antibodies were applied for 3 hours at room temperature. The nuclei were stained with propidium iodide (1:100; Molecular Probes) for 5 minutes. The cells were then cover-slipped with Vectashield mounting medium (Vector Laboratories, Burlingame, CA, USA). Samples were imaged using confocal laser scanning microscopy (Fluoview FV1000; Olympus, Tokyo, Japan).

### Immunohistochemical analysis of normal rat IVD tissues

Rat lumbar spines (vertebrae L1-L6 (*n* = 2)) were removed and fixed in 4% paraformaldehyde, followed by decalcification in 30% ethylenediaminetetraacetic acid for 28 days. The samples were embedded in paraffin and serial 5-μm cross-sections were processed and stained with safranin-O/hematoxylin and eosin (safranin-O/HE) for immunohistochemical analysis. After blocking endogenous peroxidase activity, the sections for RANK and RANKL were heated with 0.01 M citrated buffer (pH 6.0) and those for OPG were treated with proteinase K (pH 8.0). The sections were then incubated overnight at room temperature with the primary antibodies used in the monolayer culture study. The appropriate rabbit IgG (Dako), mouse IgG or goat IgG was used as the isotype control. The sections were incubated with the appropriate anti-mouse, anti-rabbit or anti-goat horseradish peroxidase (HRP)-conjugated secondary antibody (Dako). Peroxidase activity was detected with 3, 3-diaminobenzidine tetrahydrochloride (DAB; Dojindo, Kumamoto, Japan). The sections were counterstained in Mayer’s hematoxylin.

### Immunohistochemical analysis of human IVD tissues

Institutional Review Board (IRB) approval was obtained for this study. Human IVD tissues were obtained from spine surgery with informed consent from all patients. The samples were divided into different stages of degradation according to Pfirrmann’s magnetic resonance imaging (MRI) classification [18]: grade 2 (*n* = 4); grade 3 (*n* = 11); and grade 4 (*n* = 13). Grades 2 and 3 were defined as early degenerated tissue, while grade 4 was defined as advanced degenerated tissue. The samples were embedded in paraffin, and serial 5-μm sections were used for safranin-O/HE staining and immunohistochemical analysis as described. Each sample was divided into three areas: nucleus pulposus (NP), inner annulus fibrosis (iAF), and outer annulus fibrosis (oAF). With the Cell Counter plugin in Image J (National Institutes of Health), the percentages of positive staining in cells were quantified for each antibody by determining the mean percentage of immunopositive cells from five fields of microscopic images per sample at × 400 magnification (Fig. 6a).

### Statistical analysis

The data are expressed as the mean ± standard error of three independent experiments. The data were analyzed by one-way analysis of variance (ANOVA) using the between-subject factors for the different experimental groups. The post hoc analyses were performed using the Fisher protected least significant difference (PLSD) test. The evaluation of statistical differences between the groups was determined using the unpaired Student's *t* test. Significance was accepted at *p* < 0.05.

## Results

### Immunohistochemical analysis of normal rat IVD tissues

**Fig. 1:**
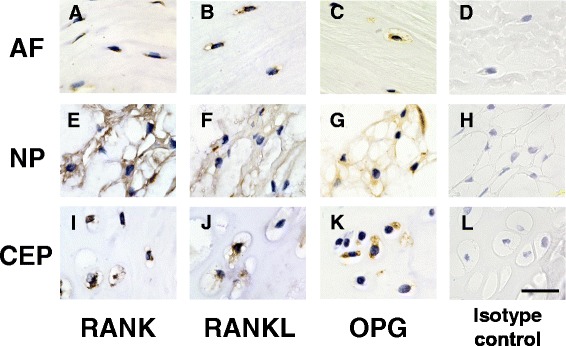
Immunohistochemical staining for receptor activator of nuclear factor kappa B (*RANK*)/RANK ligand (*RANKL*)/osteoprotegerin (*OPG*) in normal rat intervertebral disc (IVD) tissues (anulus fibrosus (*AF*) (**a**-**d**); nucleus pulposus (*NP*) (**e**-**h**); cartilaginous endplate (*CEP*) (**i**-**l**)). Paraffin-embedded sections of rat IVDs (12-week-old male rats (n = 2)) were stained with mouse monoclonal anti-RANK antibody (**a**, **e**, **i**), rabbit polyclonal anti-RANKL antibody (**b**, **f**, **j**), or goat polyclonal anti-OPG antibody (**c**, **g**, **k**). Isotype controls are shown in **d**, **h** and **l**. *Scale bar* 10 μm

Every component of the RANK/RANKL/OPG system was identified in AF, NP and CEP tissues of normal IVDs from 12-week-old rats (Fig. 1). Intense immunoreactivity to RANK, RANKL and OPG was found particularly in the NP and CEP. Cells immunoreactive to RANK, RANKL and OPG were also identified in the AF, but showed less positive immunoreactivity than in the NP. No immunoreactivity was found in isotype controls (rabbit IgG).

### Fluorescent immunohistochemical analysis of rat IVD cells

**Fig. 2:**
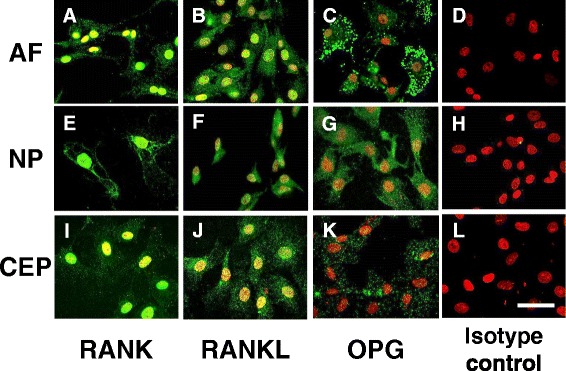
Immunohistochemical staining for receptor activator of nuclear factor kappa B (*RANK*)/RANK ligand (*RANKL*)/osteoprotegerin (*OPG*) in cultured rat anulus fibrosus (*AF*), nucleus pulposus (*NP*) and cartilaginous endplate (*CEP*) cells. All the cells were cultured in monolayer for 3 days: **a**-**d** AF; **e**-**h** NP; **i**-**l** CEP. **d**, **h**, **l** Isotype controls. Samples were imaged using confocal microscopy. Immunoreactivity (*green*) is clearly seen in the AF, NP and CEP cells. Nuclei are stained with propidium iodide (*red*). *Scale bar* 10 μm

Immunoreactivity to RANK, RANKL and OPG was clearly identified in monolayer cultures of rat AF, NP and CEP cells (Fig. 2). Confocal images revealed that immunoreactivity to RANK was primarily found in cell membranes and cytoplasm of the three types of cells. The expression of RANKL was distributed in granules in the cytoplasm of all the cells. OPG immunoreactivity was found in a spot-like distribution in the cytoplasm of all the cells. No immunoreactivity was found in the isotype controls.

### Detection of mRNA expression of *RANK*/*RANKL*/*OPG* by rat IVD and CEP cells

**Fig. 3:**
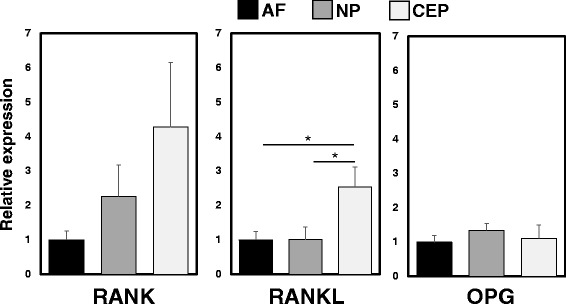
Detection of mRNA expression of *receptor activator of nuclear factor kappa B* (*RANK*)/*RANK ligand* (*RANKL*)/*osteoprotegerin* (*OPG*) in rat anulus fibrosus (*AF*), nucleus pulposus (*NP*) and cartilaginous endplate (*CEP*) cells. Significantly higher mRNA expression levels of *RANKL* were found in CEP cells than those in AF and NP cells; *p < 0.05. There were no significant differences in expression levels of *RANK* and *OPG* among AF, NP and CEP cells

The expression levels of *RANK*, *RANKL* and *OPG* were quantified using real-time PCR. mRNA expression levels of *RANK*, *RANKL* and *OPG* were clearly seen in AF, NP and CEP cells (Fig. 3). A significant, but mild increase in the mRNA expression of *RANKL* was found in CEP cells, compared to that in AF and NP cells (*p* < 0.01). However, there were no significant differences in the expression levels of *RANK* and *OPG* among the three types of cells (relative expression in the NP (vs. the AF): *RANK* 2.26 ± 0.91, *RANKL* 1.01 ± 0.37, *OPG* 1.34 ± 0.21; expression in the CEP (vs. the AF): *RANK* 4.27 ± 1.88, *RANKL* 2.54 ± 0.58, *OPG* 1.11 ± 0.39).

### The effect of IL-1β stimulation on mRNA levels of *RANK*/*RANKL*/*OPG*

**Fig. 4:**
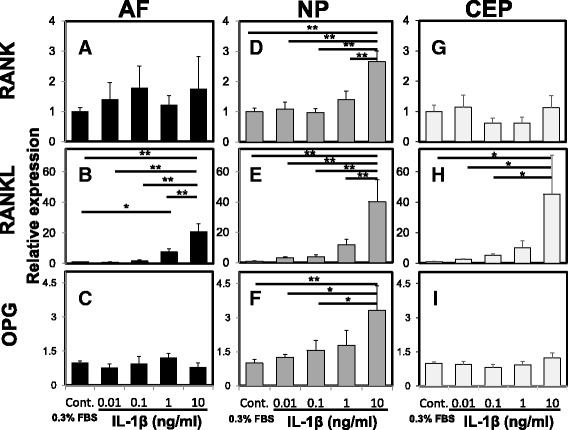
The effect of interleukin-1beta (*IL-1*β) on mRNA levels of receptor activator of nuclear factor kappa B (*RANK*)/RANK ligand (*RANKL*)/*osteoprotegerin* (*OPG*) by rat anulus fibrosus (*AF*), nucleus pulposus (*NP*) and cartilaginous endplate (*CEP*) cells. The mRNA expression levels of *RANK* (**a**, **d**, **g**), *RANKL* (**b**, **e**, **h**), and *OPG* (**c**, **f**, **i**) by AF (**a**-**c**), NP (**d**-**f**) and CEP (**g**-**i**) cells were quantified by real-time polymerase chain reaction. Stimulation of rat NP cells with IL-1β at 10 ng/mL significantly increased mRNA expression of *RANK*, *RANKL* and *OPG* compared with the control group. Stimulation of rat AF and CEP cells with IL-1β at 10 ng/mL significantly increased mRNA expression of *RANKL* compared with the control group. No significant changes in mRNA expression levels of *RANK* and *OPG* in AF and CEP cells were detected from treatment with IL-1β: *p < 0.05, **p < 0.01 vs. control. *FBS* fetal bovine serum

The mRNA level of *RANKL* was significantly upregulated by treatment with IL-1β (10 ng/mL) (relative expression (vs. control): *RANKL* in the AF 20.92 ± 5.12, *p* < 0.01; *RANKL* in the NP 40.16 ± 14.13, *p* < 0.01; *RANKL* in the CEP 45.33 ± 25.43, *p* < 0.05) (Fig. 4b, e, h). Stimulation by IL-1β (10 ng/mL) induced a significant increase in the mRNA expression of *RANK* and *OPG* in NP cells (relative expression (vs. control): *RANK* 2.66 ± 0.35, *p* < 0.01; *OPG* 3.33 ± 1.07, *p* < 0.01) (Fig. 4d, f). The mRNA expression levels of *RANK* and *OPG* in both AF and CEP cells did not significantly differ with stimulation by IL-1β (Fig. 4a, c, g, i).

### The effect of RANKL on the mRNA expression of proinflammatory cytokines and proteolytic enzymes with or without IL-1β stimulation

**Fig. 5:**
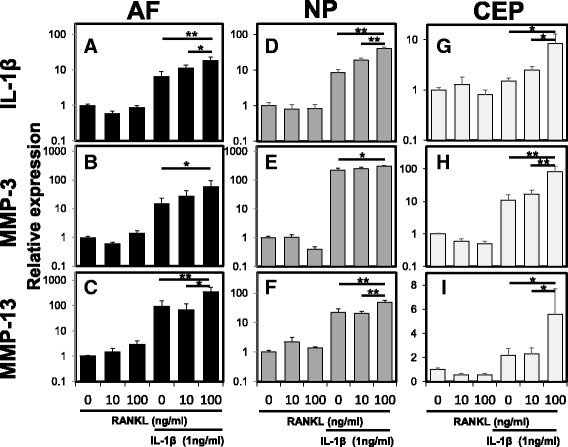
The effect of receptor activator of nuclear factor kappa B ligand (*RANKL*) on the mRNA expression of proinflammatory cytokines and proteolytic enzymes with or without recombinant human interleukin-1beta (rh*IL-1*β) stimulation. The mRNA expression levels of *IL-1*β (**a**, **d**, **g**), matrix metalloproteinase (*MMP*) -3 (**b**, **e**, **h**), and *MMP-13* (**c**, **f**, **i**) by the anulus fibrosus (*AF*) (**a**-**c**), nucleus pulposus (*NP*) (**d**-**f**) and cartilaginous endplate (*CEP*) (**g**-**i**) cells were quantified by real-time polymerase chain reaction. Treatment with RANKL in the absence of rhIL-1β did not induce a significant effect on the mRNA expression of *IL-1*β, *MMP-3* or *MMP-13* in AF, NP and CEP cells. However, the mRNA expression of *IL-1*β, *MMP-3* and *MMP-13* in AF, NP and CEP cells was significantly upregulated by the stimulation of RANKL with rhIL-1β (1.0 ng/mL): *p < 0.05, **p < 0.01 vs. RANKL 0 ng/mL + IL-1β 1 ng/mL

The mRNA expression levels of *IL-1*β, *MMP-3*, and *MMP-13* in rat AF, NP and CEP cells were quantified using real-time PCR (Fig. 5). Treatment with RANKL in the absence of rhIL-1β did not produce a significant effect on the mRNA expression of *IL-1*β, *MMP-3* and *MMP-13* by AF cells (relative expression: RANKL 100 ng/mL (vs. RANKL 0 ng/mL), *IL-1*β 0.89, *p* = 0.91; *MMP-3* 1.45, *p* = 0.98; *MMP-13* 2.92, *p* = 0.98) (Fig. 5a–c). However, the mRNA expression of *IL-1*β, *MMP-3* and *MMP-13* in AF cells was significantly upregulated by stimulation of RANKL with IL-1β (1.0 ng/mL) (relative expression: RANKL 100 ng/ml + IL-1β (vs. RANKL 0 ng/mL+ IL-1β), *IL-1*β 2.86, *p* < 0.01; *MMP-3* 3.91, *p* < 0.05; *MMP-13* 3.66, *p* < 0.01) (Fig. 5a–c).

Stimulation of NP cells by RANKL without rhIL-1β stimulation did not produce significant upregulation of mRNA expression of *IL-1*β, *MMP-3* and *MMP-13* (relative expression: RANKL 100 ng/ml (vs. RANKL 0 ng/mL), *IL-1*β 0.83, *p* = 0.96; *MMP-3* 0.40, *p* = 0.98; *MMP-13* 1.41, *p* = 0.91) (Fig. 5d–f). The mRNA expression of *IL-1*β, *MMP-3* and *MMP-13* by NP cells was significantly upregulated by stimulation with RANKL and rhIL-1β (1 ng/mL) (relative expression: RANKL 100 ng/mL + IL-1β (vs. RANKL 0 ng/mL + IL-1β), *IL-1*β 4.79, *p* < 0.01; *MMP-3* 1.38, *p* < 0.05; *MMP-13* 2.15, *p* < 0.01) (Fig. 5d-f).

Similarly, stimulation of CEP cells by RANKL without rhIL-1β stimulation did not induce significant changes in mRNA expression of *IL-1*β, *MMP-3* and *MMP-13* (relative expression: RANKL 100 ng/mL (vs. RANKL 0 ng/mL); *IL-1*β 0.81, *p* = 0.95; *MMP-3* 0.50, *p* = 0.99; *MMP-13* 0.56, *p* = 0.91) (Fig. 5g–i). The mRNA expression of *IL-1*β, *MMP-3* and *MMP-13* by CEP cells was significantly upregulated by stimulation with RANKL and rhIL-1β (1 ng/mL) (relative expression: RANKL 100 ng/mL + IL-1β (vs. RANKL 0 ng/mL + IL-1β); *IL-1*β 5.68, *p* < 0.05; *MMP-3* 7.49, *p* < 0.01; *MMP-13* 2.54, *p* < 0.05) (Fig. 5g–i).

### Expression of the RANK/RANKL/OPG system in human IVD tissues with different stages of degeneration

**Fig. 6:**
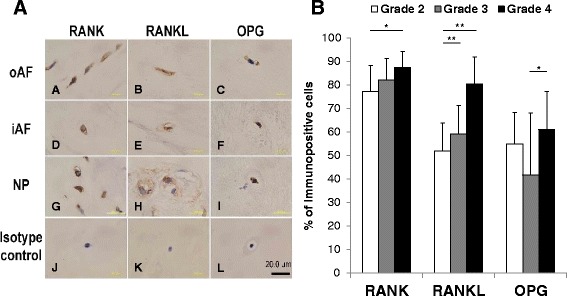
Immunohistochemical staining for the receptor activator of nuclear factor kappa B (*RANK*)/RANK ligand (*RANKL*)/osteoprotegerin (*OPG*) system in human intervertebral disc (IVD) tissues. **a** Paraffin-embedded sections of human IVD tissues (outer anulus fibrosus (*oAF*) (*A*-*C*); inner anulus fibrosus (*iAF*) (*D*-*F*); nucleus pulposus (*NP*) (*G*-*I*) were stained with mouse monoclonal anti-RANK antibody (*A*, *D*, *G*), rabbit polyclonal anti-RANKL antibody (*B*, *E*, *H*) or goat polyclonal anti-OPG antibody (*C*, *F*, *I*). *J*, *L* Isotype controls. *Scale bar* 20 μm. **b** Percentage of cells immunopositive for RANK, RANKL and OPG in different grades of disc degeneration: *p < 0.05, **p < 0.01

The total percentage of cells immunopositive for RANK was significantly higher in grade 4 tissues compared to that in grade 2 tissues (mean percentage of immuroreactive cells: grade 2, 77.2 ± 11.0; grade 3, 82.1 ± 9.2; grade 4, 87.5 ± 6.7; *p* < 0.05 vs. grade 2) (Fig. 6b). The percentage of cells with a positive stain in the NP region was significantly higher in grade 4 tissues compared to that in grade 3 tissues (*p* < 0.05). There were no significant differences between the different grades of degeneration in the iAF and oAF regions.

The total percentage of cells immunoreactive for RANKL was significantly higher in grade 4 tissues compared to those in grade 2 and grade 3 tissues (mean percentage of immuroreactive cells: grade 2, 51.9 ± 11.8; grade 3, 59.2 ± 12.1; grade 4, 80.4 ± 11.5; *p* < 0.01 vs. grade 2 and grade 3, respectively, Fig 6b). Grade 3 and grade 4 tissues had significantly higher percentages of cells immunoreactive for RANKL in the oAF region than grade 2 tissues (*p* < 0.05, respectively). In the iAF region, grade 4 tissues had a significantly higher percentage of cells with a positive stain than those in grade 2 and grade 3 tissues (*p* < 0.01, respectively). The percentage of positive stains in the NP region was significantly greater in grade 4 than in grade 3 tissues (*p* < 0.05).

The total percentage of cells with positive staining for OPG was significantly higher in grade 4 tissues compared to that in grade 3 tissues (mean percentage of immuroreactive cells: grade 2, 54.9 ± 13.5; grade 3, 41.7 ± 26.5; grade 4, 61.1 ± 16.1; *p* < 0.05 vs. grade 3, Fig. 6b). Within the total amount of immunoreactive cells, grade 4 tissues had a significantly greater percentage of positively stained cells compared to grade 3 tissues in the NP region (*p* < 0.05). There were no significant differences between the different grades of tissue in the iAF and oAF regions.

## Discussion

The present study demonstrated that expression of the RANK/RANKL/OPG system was confirmed both at mRNA and protein levels in the rat IVD, including the in CEP. Quantitative mRNA analysis identified a similar mRNA expression level of the *RANK*/*RANKL*/*OPG* system by AF, NP and CEP cells. To examine the effect of the RANK/RANKL/OPG system on matrix metabolism, exogenous IL-1β and/or RANKL were administered to rat IVD and CEP cells. The expression of *RANKL* was regulated by stimulation with IL-1β by rat AF, NP and CEP cells. Although catabolic factors, such as *IL-1*β, *MMP-3* and *MMP-13*, were not stimulated by RANKL alone, the gene expression was significantly enhanced by stimulation with RANKL in the presence of IL-1β.

An immunohistochemical analysis recently reported by Mackiewicz et al. [15] showed that RANK, RANKL and OPG are expressed in degenerated human AF tissue. Gruber et al. [16] report that the gene expression of RANKL in the human disc is significantly higher in degenerated IVDs compared to normal IVDs. Increased expression of OPG has also been identified in degenerated human IVD tissues compared to healthy IVDs [17]. The results of these previous studies and our immunohistochemical human IVD study suggest that the expression of the RANK/RANKL/OPG system identified in human IVDs may be associated with the progression of disc degeneration. The results of the present study demonstrate, for the first time, that each component of the RANK/RANKL/OPG system was constitutively expressed in normal rat AF and NP cells both at mRNA and protein levels, and also in those tissues at the protein level.

In this study, to mimic the micro-environment that may occur in degenerated IVDs [2], rat AF and NP cells were cultured in the presence of IL-1β. The mRNA expression of *RANKL* was significantly and dose-dependently upregulated by both rat AF and NP cells by stimulation with IL-1β. This result supports a previous report showing that the gene expression of *RANKL* was higher in degenerated discs than in normal discs [16]. Importantly, in this study, the response to IL-1β stimulation was greater in NP cells than in AF cells. On the other hand, IL-1β produced significant, but minor, stimulation of the gene expression of *RANK* and *OPG* by NP cells, but not AF cells. These results indicate that, among the three components of the RANK/RANKL/OPG system, the expression of *RANKL* was most strongly stimulated by IL-1β.

Proinflammatory cytokines, such as TNF-α, IL-1β and IL-6, have been implicated in the pathogenesis of inflammatory osteolysis (osteoclastgenesis), both directly and indirectly [19]. These proinflammatory cytokines markedly stimulate osteoblasts or stromal cells to produce RANKL, with a minor effect on the release of OPG [19–22]. These molecular changes are considered to be among the factors leading to the imbalance of homeostasis of the bone matrix in inflammatory bone-erosive diseases. Interestingly, these responses of proinflammatory cytokines on the expression of *RANKL* and/or *OPG* by osteoblasts were similar to those on rat IVD cells shown in this study. This suggests that proinflammatory cytokines may have a role in regulating the balance between the expression of *RANKL* and *OPG*, not only by the osteoblastic phenotype, but also by the chondrogenic phenotype, including IVD cells.

Although our study showed no, or only minor, effects of IL-1β on the expression of *OPG* by rat IVD cells, other studies have reported positive correlation between the grade of degeneration of human IVD tissues and OPG levels in IVD tissues [17] and serum [23]. Micro-environmental differences between human IVD tissues and rat cells cultured in vitro might also be responsible for this discrepancy between our study and others.

To examine the effect of RANKL on matrix metabolism, rat IVD cells were treated with exogenous RANKL. The gene expression of catabolic molecules, such as *IL-1*β, *MMP-3* and *MMP-13* by both rat AF and NP cells was not stimulated by RANKL alone. Similar results have been reported from previous studies on human articular chondrocytes [13, 14] showing that exogenous RANKL does not activate NF-κ B or induce the expression of genes encoding catabolic mediators. Kwan Tat et al. [14] speculated that a low percentage of RANK-positive sub-populations in human articular chondrocytes may be related to the mechanism for this lack of effect by RANKL stimulation. Multiple isoforms of RANK truncated in their intracellular domain have been reported [24]. One of these isoforms of RANK (*TNFRSE11A_e5a*) was recently found to have low affinity for the RANKL capable of activating NF-κB [25]. These previous studies suggest that the protein level and/or the character of isoform types may also affect RANKL signaling by rat IVD cells.

In contrast, the results of our study showed that the mRNA expression of *IL-1*β, *MMP-3* and *MMP-13* by both rat AF and NP cells was significantly upregulated by exogenous RANKL in the presence of IL-1β. This result led us to speculate that proinflammatory cytokines, such as IL-1β, may have the potential to activate the RANKL signaling pathway by affecting the protein level and/or the character of isoform types of RANK, which is associated with the NF-κB signaling pathway. Although RANKL stimulation alone did not influence the expression of catabolic factors in normal IVDs, these catabolic factors may be accelerated by stimulation with RANKL in the proinflammatory cytokine-rich micro-environment found in degenerating IVDs.

The CEP is a layer of hyaline cartilage found between IVD tissues and the vertebral body, which plays an important role in regulating nutrient transport and metabolic exchange [26]. The biochemical characteristics of the extracellular matrix of the CEP are similar to those of IVD tissues, and during IVD degeneration, the CEP also shows degenerative changes, including deceases in proteoglycan and collagen content and upregulation of proinflammatory cytokines and proteolytic enzymes, hallmarks similar to those of IVD degeneration [27, 28]. Therefore, we additionally examined the expression of the RANK/RANKL/OPG system in the rat CEP, and also evaluated the effect of RANKL and rhIL-1β on the matrix metabolism of rat CEP cells. mRNA and protein expression of the RANK/RANKL/OPG system was also found in rat CEP cells, and the response of rhIL-1β and/or RANKL stimulation on rat CEP cells showed a tendency similar to that found in rat IVD cells.

To further test the hypothesis, we have additionally examined the expression of the RANK/RANKL/OPG system in human IVD tissues with different stages of degeneration. The present study showed there was a general trend for a higher presence of RANK/RANKL/OPG-positive cells in human IVD tissues in an advanced stage of degeneration compared to that of an early stage of degeneration. There was a significant increase in the expression in the NP region for all three proteins of the RANK/RANKL/OPG system. RANKL differed from the other proteins in that there was a significant increase in expression in all three areas in advanced degenerated tissues compared to those in the early degenerated tissues. The results of this study suggest that the expression of the RANK/RANKL/OPG system, especially RANKL, could be associated with the progression of disc degeneration.

## Conclusions

The results of this study demonstrated that the RANK/RANKL/OPG system is present in the rat IVD, including the CEP. Among the components of the RANK/RANKL/OPG system, the gene expression of *RANKL* was remarkably upregulated by treatment with IL-1β in vitro by rat AF, NP and CEP cells. We can speculate that the expression of RANKL is enhanced in the proinflammatory-rich micro-environment of the degenerated IVD; thus, the RANK/RANKL/OPG system may play a part in the complex molecular mechanism of IVD degeneration by interactions with proinflammatory cytokines. These molecular events would also be applicable for the pathogenesis of CEP degeneration. Additionally, the significant increase in expression of the RANK/RANKL/OPG system in the advanced stage of degenerated human IVD tissues suggests that its expression would be associated with the progression of disc degeneration.

## Abbreviations

AF: Anulus fibrosus
ANOVA: Analysis of variance
CEP: Cartilaginous endplate
FBS: Fetal bovine serum
Fisher PLSD test: Fisher protected least significant difference test
HE: Hematoxylin and eosin
HRP: Horseradish peroxidase
iAF: Inner annulus fibrosis
IgG: Immunoglobulin G
IL-1β: Interleukin-1 beta
IRB: Institutional Review Board
IVD: Intervertebral disc
MMP: Matrix metalloproteinase
NF-κB: Nuclear factor kappa B
NP: Nucleus pulposus
oAF: Outer annulus fibrosis
OPG: Osteoprotegerin
PCR: Polymerase chain reaction
RANK: Receptor activator of nuclear factor kappa B
RANKL: Receptor activator of nuclear factor kappa B ligand
rhIL-1β: Recombinant human IL-1β
rRNA: Ribosomal RNA
TNF-α: Tumor necrosis factor-alpha
TRAF: Tumor necrosis factor receptor-associated factor
